# Supplementary material for: Identification of photoperiod-induced specific miRNAs in the adrenal glands of Sunite sheep (Ovis aries)
Source: Front Vet Sci. 2022 Jul 22;9:888207. doi: 10.3389/fvets.2022.888207 (PMC9354845; doi:10.3389/fvets.2022.888207)
Supplement: Supplementary file 1 [file Data_Sheet_1.ZIP › Supplementary file/miRDeep2 file.zip!/miRDeep2 file/Novel_Structure/LP42A2/Novel_530.pdf]

Provisional ID	:	NC_019466.2_17129
Score total	:	60.4
Score for star read(s)	:	3.9
Score for read counts	:	53.1
Score for mfe	:	1.7
Score for randfold	:	1.6
Score for cons. seed	:	
Total read count	:	116
Mature read count	:	113
Loop read count	:	0
Star read count	:	3

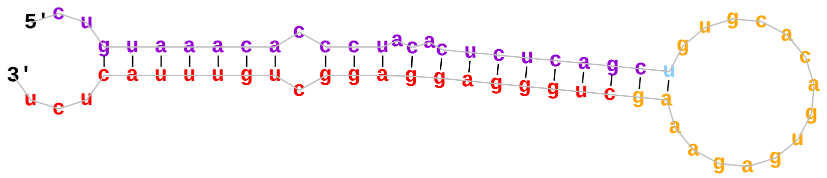

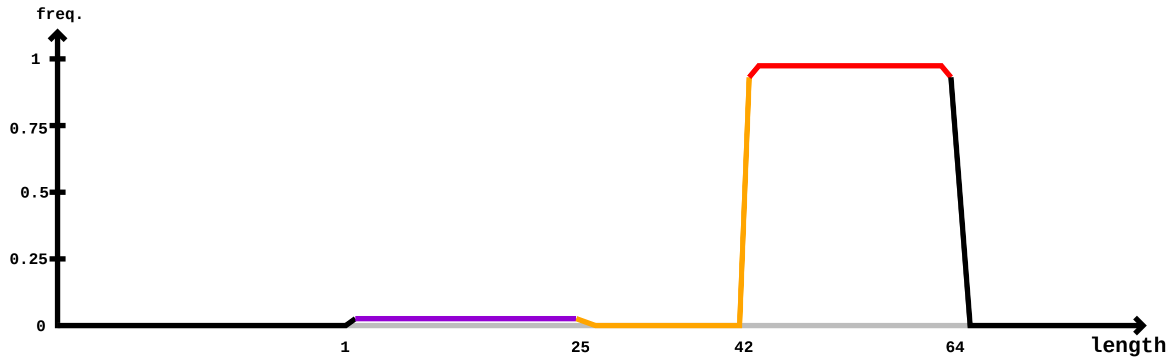


```
                                 Star                                         Mature
5'- ccgccccagagagcacugagggccagauacuguaaacacccuacacucucagcugugcacagugagaaagcugggaggaggcuguuuacucucucugccuuggaagucaac   -3'    obs
    ccgccccagagagcacugagggccagauacuguaaacacccuacacucucagcugugcacagugagaaagcugggaggaggcuguuuacucucucugccuuggaagucaac          exp
    ..(((((((......))).))))((((....(((((((.(((.(.(((((((((..............))))))))))))).)))))))....))))..............   reads   mm   sample
    .............................cuguaaacacccuacacucucagc..........................................................   2       0    seq
    .............................cuguaaacacccuacacucucagcu.........................................................   1       0    seq
    ......................................................................cugggaggaggcuguuuacuc....................   5       0    seq
    ......................................................................cugggaggaggcuguuuacucu...................   102     0    seq
    ......................................................................cugggaggaggcuguuuacucuc..................   1       0    seq
    .......................................................................ugggaggaggcuguuuacucu...................   5       0    seq
```
